# Synergistic Antiviral Activity of Xanthan Gum and Camostat Against Influenza Virus Infection

**DOI:** 10.3390/v17030301

**Published:** 2025-02-21

**Authors:** Kyeunghwa Chun, Yujeong Na, Byeongyong Kim, Dongjin Lee, Jongseo Choi, Gwanyoung Kim, Sokho Kim, Min-Soo Kim

**Affiliations:** 1 Daewoong Pharmaceutical Co., Ltd., 72, Dugye-ro, Pogok-eup, Cheoin-gu, Yongin-si 17028, Gyeonggi-do, Republic of Korea; khcheon086@daewoong.co.kr (K.C.); 2210494@daewoong.co.kr (Y.N.); 2210565@daewoong.co.kr (B.K.); leedj@daewoong.co.kr (D.L.); 2210302@daewoong.co.kr (J.C.); pharmrich@daewoong.co.kr (G.K.); 2 College of Pharmacy, Pusan National University, 2, Busandaehak-ro 63beon-gil, Geumjeong-gu, Busan 46241, Republic of Korea; 3 Major of Biohealth Regulatory Science, School of Liberal Studies, Kunsan National University, 558 Daehak-ro, Gunsan 54150, Jeollabuk-do, Republic of Korea

**Keywords:** influenza A virus, xanthan gum, camostat, antiviral effect

## Abstract

Influenza A virus (IAV) is a major cause of respiratory infections worldwide. Current preventive measures, though effective for decades, face limitations due to the continuous evolution of viral strains and challenges in targeting specific viral proteins. In this study, we conducted in vivo screenings to evaluate the antiviral properties of various promising polymers to overcome the limitations of current virus infection prevention strategies. Subsequently, we performed detailed physiological and pathological assessments over an extended infection period. In the animal experiments regarding weight loss, a key symptom of viral infection, the group treated with xanthan gum (XG) showed significant suppression of weight loss compared to the untreated group starting from 3 DPI. Throughout the experiment, the treated group maintained a body weight similar to that of the uninfected group. In the virus titration and lung tissue pathology analysis, the group treated with the test substance showed a significantly lower viral load and tissue pathology results closer to normal compared to the untreated group. Additionally, we conducted in vitro combination treatment experiments to evaluate the antiviral ability of XG in conjunction with camostat, a previously known TMPRSS2 inhibitor. The results demonstrated that in the combination-treated groups, XG and camostat exhibited significantly higher cell viability at lower concentrations compared to the single-treatment groups for influenza A H1N1, A H3N2, and B type. These results indicate that XG possesses potential capabilities in inhibiting respiratory viruses and may be utilized in conjunction with existing antiviral treatments.

## 1. Introduction

Influenza viruses are primarily transmitted through aerosols, spreading through the respiratory tract and causing significant morbidity and mortality worldwide. Influenza viruses frequently cause outbreaks, become endemic, and ultimately lead to pandemics, affecting an estimated 5–10% of adults and 20–30% of children annually [1]. The current strategies for controlling influenza virus-causing epidemics rely heavily on vaccination and treatment with small-molecule drugs [2]. Since 2010, the Centers for Disease Control and Prevention (CDC) has recommended routine annual influenza vaccination for all individuals over 6 months of age without contraindications [3]. However, these approaches face significant challenges. First, the rapid mutation of the virus necessitates the annual reformulation of vaccines to combat new variants effectively [4]. Additionally, the emergence of drug-resistant influenza strains reduces the efficacy of existing treatments. Second, the development of new antiviral drugs is a lengthy and costly process that requires a deep understanding of potential target proteins at the molecular and structural levels, as many commercial drugs struggle with low binding affinity to their targets [5].

Therefore, to address the ongoing emergence of viral variants and the complexities, high costs, and delays associated with antiviral drug development, we explored alternative formulations that can prevent the early threats of respiratory viruses. As a result, we conducted a study utilizing multivalent polymeric inhibitors. Recently, it has been confirmed that carrageenan oligosaccharides, antioxidant polysaccharides extracted from red seaweed, exhibit antiviral activity against respiratory viruses, including influenza viruses [6,7]. We investigated multivalent polymeric inhibitors and selected fucoidan, xanthan gum (XG), bentonite, and hydroxypropyl methylcellulose (HPMC) for screening purposes.

Fucoidan, a heterogeneous class of polysaccharides found in the extracellular matrix and cell wall of brown seaweed (Phaeophyta), is composed of a sulfated backbone with diverse sugar monomers [8]. The diverse biochemical composition of fucoidan has attracted scientific interest for its potential bioactivities, including antioxidant, antihyperglycemic, anti-inflammatory, and anticoagulant effects [9]. Similarly, XG, a negatively charged polymer with intrinsic adjuvanticity, contains a mannose moiety that acts as a toll-like receptor 2 agonist [10,11]. Due to its unique biochemical and structural properties advantageous for effective drug delivery systems, XG has a high potential for pharmaceutical applications [12]. Bentonite is a natural clay primarily composed of montmorillonite, along with other minerals such as feldspar, calcite, and quartz [13]. Due to its high cation exchange capacity, extensive surface area, ability to form thixotropic gels with water, and capacity to absorb large amounts of gas, bentonite has numerous medicinal applications [14]. HPMC belongs to the group of cellulose ethers in which hydroxyl groups have been substituted with one or more of the three hydroxyl groups present in the cellulose ring [15]. It has been reported to inhibit virus infection in vitro and cause sustained release of highly soluble drugs [16]. Despite their potential to enhance cellular immune responses and function as antiviral agents, the effectiveness of polymeric inhibitors in suppressing influenza virus infections remains largely unexplored. Additionally, we aimed to determine whether the multivalent polymeric inhibitors we identified exhibit stronger effects when combined with previously developed antiviral agents. To investigate this, we evaluated the effects of co-treatment with camostat mesylate (hereafter, camostat), which has viral entry inhibition capabilities [17,18], alongside the multivalent polymeric inhibitors selected in our screening process.

In this study, we aimed to evaluate the effects of various polymers on influenza virus infections. Through in vivo screening of the aforementioned polysaccharides, we identified potential polymers against the influenza virus and selected the most promising candidate. XG, the selected polymer, was evaluated for its antiviral activity at various concentrations through in vivo experiments. Additionally, we conducted in vitro experiments to assess the potential of combination treatment with camostat. These results confirm the potential of xanthan gum as an effective antiviral agent, with its efficacy boosted by camostat. Our findings provide a valuable foundation for the development of new antiviral strategies and highlight the potential pharmaceutical applications of antiviral polysaccharides.

## 2. Materials and Methods

### 2.1. Viruses

Flu influenza virus strain FluV/A/PR8/34/H1N1 was used for all in vivo experiments, while FluV/A/PR8/34/H1N1, FluV/A/Hong Kong/H3N2, and influenza B strain FluV/B/Lee were used in the in vitro experiments. All viruses were propagated via inoculation into the allantoic sac of 9-day-old embryonated chicken eggs using standard procedures [19]. After passage in 9–10-day-old embryonated eggs, the allantoic fluid was harvested and stored at −70 °C. The viral titer was measured 48 h post-inoculation and calculated as plaque-forming units per milliliter (PFU/mL) according to the method described in [20]. For in vivo studies, the virus diluted to a concentration of 1 LD50 (total 30 µL) was administered intranasally to mice through the left nasal cavity. The 1 LD50 was determined to be 100 PFU/mL.

### 2.2. Mouse Preparation and Handling

A total of 248 six-week-old female C57BL/6 mice were purchased from Samtaco, Inc. (Kyunggi, Republic of Korea) for use in the in vivo experiments. The mice were housed in filter-top microisolator cages and maintained under controlled conditions: 23 °C, 55% humidity, and a 12 h light/dark cycle. They were provided with water and feed ad libitum. During the first seven days, we monitored the mice for health status, including feeding behavior and growth, and selected 236 healthy mice (average body weight 20 g) for further analyses. Body weight, lethality, and clinical symptoms were monitored daily from 0 to 7 days post-infection (DPI). All animal experiments were approved by the Institutional Animal Care and Use Committee (IACUC) of KNOTUS and conducted in compliance with Institutional Biosafety Committee guidelines and the ARRIVE guidelines (Approval code: KNOTUS IACUC 22-KE-0363, KNOTUS IACUC 22-KE-0709).

### 2.3. Polymer Treatment

All drugs and substances were administered to the mice via the nasal cavity, except for oseltamivir, which was given orally. Specifically for HPMC, XG, and fucoidan, carrageenan was sprayed directly into the nasal cavity using a syringe designed for spray dispersion, while bentonite was administered via micropipette. All test substances were dissolved in D.W. before being administered to the animals. In all animal experiments, the test substances were administered before viral infection and were given twice daily at 8 h intervals. The single-dose amount and volume per administration for the multivalent polymeric inhibitor screening experiment (MPE) are listed in Table 1, while the dose-dependent antiviral inhibition experiment (DDE) for XG is detailed in Table 2.

### 2.4. Experimental Design for the Two In Vivo Studies

MPE is conducted to compare the potential in vivo antiviral effects of XG, fucoidan, bentonite, and HPMC using C57BL/6 female mice over an extended period of influenza virus infection from 0 DPI to 7 DPI. A total of 124 mice were divided into nine groups (G1 to G9) (Table 1). G1 served as the normal control group, receiving no antiviral drug or polymer. G2 was the infection control, treated with water similarly to the test groups, while G3 acted as the vehicle control, receiving a placebo. For positive controls, G4 and G5 were treated with oseltamivir (Tamiflu) and λ-carrageenan, respectively, as both oseltamivir and lambda-carrageenan are established antiviral agents [21,22,23]. The test groups (G6 to G9) were treated with HPMC, XG, bentonite, and fucoidan, respectively. No side effects or medical complications were observed during the trials, indicating that treatment with these polymers does not result in adverse health outcomes.

DDE is conducted to evaluate the efficacy via a dose-dependent manner of XG. A total of 112 mice were divided into seven groups (G1 to G7) (Table 2). G1 served as the normal control with no antiviral treatment, while G2 and G3 functioned as infection and vehicle controls, respectively, as in the previous screening. λ-Carrageenan was selected as the positive control due to its consistent antiviral efficacy [7,23]. The test groups (G5, G6, and G7) received low (0.005 mg/head), medium (0.01 mg/head), and high (0.02 mg/head) doses of XG, respectively.

### 2.5. Histopathological Analysis

Autopsies were performed at 3, 5, and 7 DPI on five mice from each group, except for groups G1 of MPE and G1 of DDE, where all mice were autopsied at 5 DPI. During the autopsy, swab specimens were collected for viral titer measurements and stored at −70 °C until analysis. H1N1-infected lung tissues were fixed in 4% formalin, dehydrated using graded ethanol, embedded in paraffin, sectioned into 4 µm slices, and stained with hematoxylin and eosin (H&E; Thermo Scientific/Merck, Waltham, MA, USA). Lung damage was scored based on an established system [24] as follows: minimal, scattered inflammatory cells in the parenchyma; mild, aggregated inflammatory cells in <1/3 of the parenchyma; moderate, aggregated inflammatory cells in 1/3 to 2/3 of the parenchyma; and severe—aggregated inflammatory cells in >2/3 of the parenchyma.

### 2.6. Virus Quantification

RNA was extracted from the lung and from the swab samples collected during the autopsy, following the protocol described previously [25]. The expression levels of the *M2* and *polPA* genes were then quantified using standard curve-based measurements. Briefly, tissues of the lung and nasal cavity harvested from mice were employed for the extraction of total RNAs using WizolTM Reagent (wizbiosolutions, Seongnam, Republic of Korea). Prepared RNAs were subjected to real-time qRT-PCR using a CFX96 Real-Time PCR Detection system (Bio-Rad Laboratories, Hercules, CA, USA). Following reverse transcription of total RNA with High-Capacity cDNA Reverse Transcription Kits (Applied Biosystems, Foster, CA, USA), the reaction mixture (20 μL total) contained 2 μL of template cDNA, 10 μL of 2× Premix Ex Taq, and 200 nM primers and probe (*polPA* gene: forward primer, 5′-CGG TCC AAA TTC CTG CTG A-3′; reverse primer, 5′-CAT TGG GTT CCT TCC ATC CA-3′; probe [HEX]5′-CCA AGT CAT GAA GGA GAG GGA ATA CCG CT-3′[BHQ1]; *M2* gene: forward primer 5′-CTT CTA ACC GAG GTC GAA ACG TA-3′; reverse primer 5′-GGT GAC AGG ATT GGT CTT GTC TTT A-3′; probe [FAM]5′-TCA GGC CCC CTC AAA GCC GAG-3′[BHQ1]). These reactions were denatured at 95 °C for 30 s and then subjected to 45 cycles of 95 °C for 5 s and 60 °C for 20 s. A control sample lacking template DNA was run with each assay. All measurements were performed at least in duplicate to ensure reproducibility. The authenticity of the amplified product was determined by melting curve analysis. All data were analyzed using Bio-Rad CFX Manager, version 2.1 analysis software (Bio-Rad Laboratories). Viral burden was expressed by the copy number of viral RNA per nanogram of total RNA and milliliter (mL) of NALF after calculating the absolute copy number of viral RNA in comparison with the standard cDNA template of viral RNA.

### 2.7. In Vitro Analysis for Antiviral Effects

Madin-Darby canine kidney (MDCK; purchased from ATCC) cells were infected with each virus at a multiplicity of infection (MOI) of 0.001 in 96-well plates (3 × 10^4^ cells/well) and incubated at 37 °C for 1 h. After incubation, the cells were washed with 1× PBS and treated with the test substances in MEM containing 2 µg/mL TPCK-trypsin. The test substances were prepared as a 100% stock solution by dissolving 1000 μg/mL of XG and 1400 μg/mL of camostat together in distilled water. The prepared 100% stock solution was first diluted 10-fold with cell culture medium and then serially diluted to final concentrations of 3.3, 1.1, 0.4, 0.1, 0.04, 0.01, 0.005, 0.002, and 0.001, which were used for the experiments. The cells were then incubated at 37 °C for 3 days. Cell viability was assessed using the MTT assay (3-(4,5-dimethylthiazol-2-yl)-2,5-diphenyltetrazolium bromide) following the manufacturer’s instructions (Thermo Fisher Scientific, Waltham, MA, USA). The CC50 (cytotoxic concentration 50%) and EC50 (the concentration required to inhibit 50% of viral activity) values were calculated using GraphPad Prism 6 software (GraphPad, La Jolla, CA, USA).

### 2.8. Statistical Analysis

Parametric or non-parametric statistical procedures were used to compare the mean values of the parameters across groups. For parametric comparisons, one-way ANOVA followed by Dunnett’s multiple comparison test was applied. In cases where non-parametric methods were required, the Kruskal–Wallis rank-sum test and Mann–Whitney U test were used. For the experimental results, parametric statistical procedures were used for analyzing weight changes and viral titers, while non-parametric methods were applied for histopathological analysis. We used GraphPad Prism 6 software to analyze and summarize the results. Statistical significance was determined at *p* < 0.05.

## 3. Results

### 3.1. Time-Course Evaluation of Antiviral Effects of Polymers: XG as a Prominent Candidate

**Figure 1:**
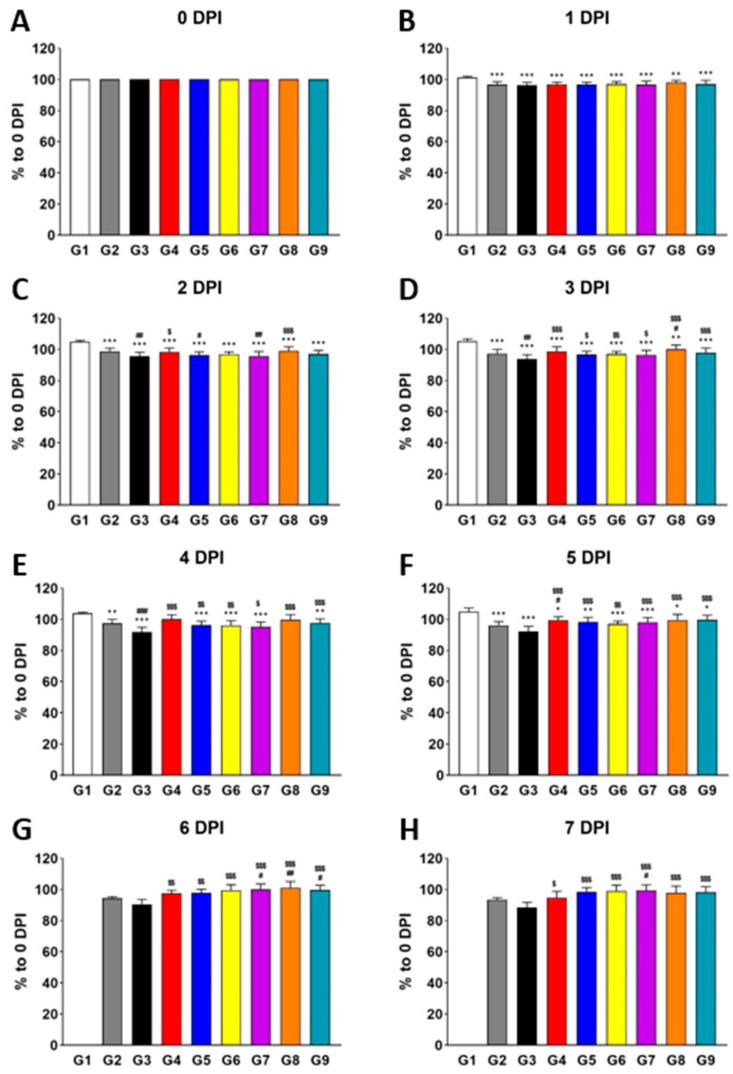
Treatment with individual polymers (HPMC, XG, bentonite, and fucoidan) increases body weight during influenza virus infection in vivo. Bar graphs represent the relative body weight of each group compared to 0 DPI (**A**), measured at 1 DPI (**B**), 2 DPI (**C**), 3 DPI (**D**), 4 DPI (**E**), 5 DPI (**F**), 6 DPI (**G**), and 7 DPI (**H**). Significance levels are indicated as follows: *** *p* < 0.001 and ** *p* < 0.01 compared to G1 (normal control); ^###^ *p* < 0.001 and ^##^ *p* < 0.01 compared to G2 (infection control); ^$$$^ *p* < 0.001, ^$$^ *p* < 0.01, and ^$^ *p* < 0.05 compared to G3 (vehicle control). Data are presented as means ± s.d.; n = 15 biological replicates per group, except for G1, where four mice were analyzed. Detailed information on experimental groups is provided in Table 1.

To thoroughly assess the potential antiviral effects of the polymers, we performed daily monitoring of virus titer, body weight, and histopathological phenotypes over an 8-day period following virus infection (DPI). This time-course monitoring enabled us not only to quantify the antiviral effects but also to capture the temporal dynamics of these responses in vivo (Figure 1). As early as 1 DPI, the body weight of all infected groups (G2–G9) was significantly lower than that of the normal control (G1), indicating the infection’s impact on body weight. By 2 DPI, G3 (vehicle control) exhibited a further decrease in body weight compared to G2, supporting the known background effects of placebo [26,27] and establishing G3 as a suitable vehicle control for comparison with the test groups. Next, we focused on comparing the positive control groups (G4 and G5) and test groups (G6–G9) with G3. As expected, due to the known antiviral properties of oseltamivir and λ-carrageenan [7,28], G4 and G5 showed significantly higher body weight than G3 of MPE starting at 2 DPI. Interestingly, the test groups (G6, G7, G8, and G9 of MPE) also demonstrated significantly higher body weight than G3 of MPE, with this effect becoming evident as early as 3 DPI. This suggests that the polymers have a rapid influence on body weight during infection.

**Figure 2:**
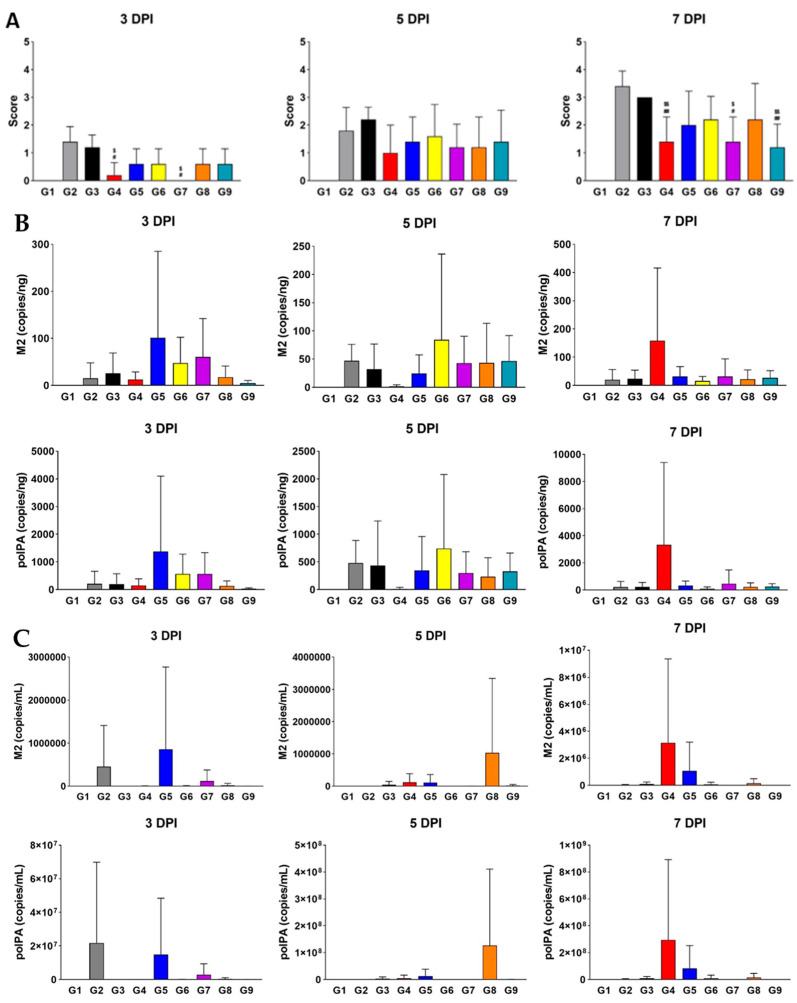
Treatment with XG significantly suppresses pathological symptoms during influenza virus infection. (**A**) Bar graphs represent the relative histopathological scores for each group (G2 to G9). (**B**,**C**) Virus titer quantification in the lung (**B**) and nasal cavity (**C**). Significance levels are indicated as follows: ^##^ *p* < 0.01 and ^#^ *p* < 0.05 compared to G2 (infection control); ^$$^ *p* < 0.01 and ^$^ *p* < 0.05 compared to G3 (vehicle control). Data are presented as means ± s.d.; n = 5 biological replicates per group. Detailed information on the experimental groups is provided in Table 1.

To visualize tissue-level damages caused by influenza virus infection, we performed H&E staining of the lung tissues from each group (Figure 2A). Our histopathological analysis revealed that the inflammation scores for G4 of MPE and G7 of MPE were significantly lower than those of G2 of MPE and G3 of MPE at 3 DPI. By 7 DPI, G4, G7, and G9 of MPE showed significantly lower scores compared to both G2 and G3 of MPE. To further investigate the effects of the polymers in suppressing viral replication, we conducted virus titer analyses in the lung and nasal cavity using the well-established PCR method to quantify *M2* and *polPA* gene copies [25]. While no significant differences were observed in the lung tissue between G3 and the test groups (G6, G7, G8, and G9 of MPE), the nasal cavity showed a clear decreasing trend in both *M2* and *polPA* copies, particularly at 5 DPI and 7 DPI (Figure 2B). Overall, our data suggest that while all polymer-treated groups generally exhibited improved body weight during infection, XG had the most pronounced antiviral effects, both physiologically (body weight) and pathologically (H&E staining and viral titters), making it the most promising candidate as an antiviral polymer.

### 3.2. Various Dosages of XG Enhance Antiviral Efficacy In Vivo

Since our in vivo screening identified XG as a promising antiviral polymer, we aimed to test various dosages of XG in suppressing viral infection in vivo. To achieve this, we conducted another experiment using a range of XG concentrations (low, medium, and high).

**Figure 3:**
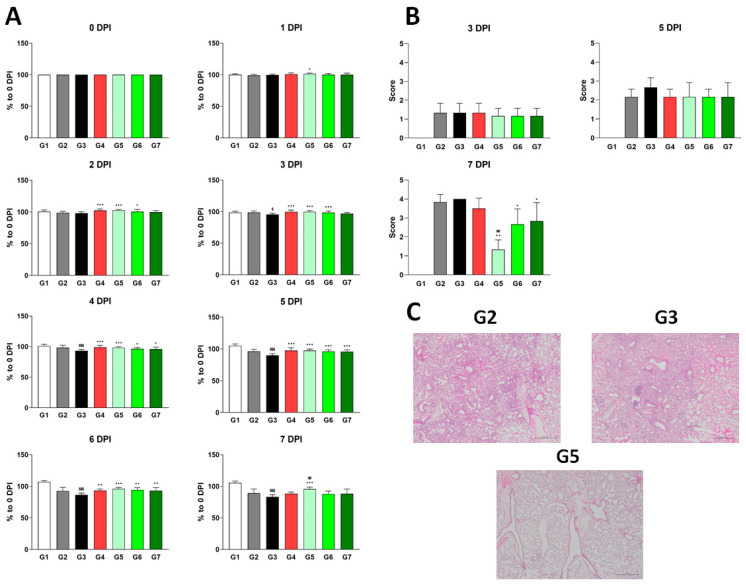
Different dosages of XG exhibit suppressive effects on influenza virus infection in vivo. (**A**) Body weight increases following XG treatment at various dosages. Significance levels are denoted as follows: $$$ *p* < 0.001 and $ *p* < 0.05 compared to G1 (normal control); ## *p* < 0.01 compared to G4 (positive control); ***, **, and * indicate *p* < 0.001, *p* < 0.01, and *p* < 0.05, respectively, compared to G3 (vehicle control). (**B**) Bar graphs represent the relative histopathological scores for each group. (**C**) Representative histopathological images from G2, G3, and G5. Data are presented as means ± s.d.; n = 18 biological replicates per group, except for G1, where four mice were analyzed. Detailed information on the experimental groups is provided in Table 2.

Consistent with our earlier findings, no side effects or medical complications were observed, confirming the safety of XG. Notably, by 1 DPI, G5 (low dose) exhibited a significant increase in body weight relative to the vehicle control (G3), which only showed a body weight increase at 2 DPI (Figure 3A). This suggests that XG exerts its effects earlier than λ-carrageenan, even at the low dose. G6 (medium dose) and G7 (high dose) showed significant body weight increases by 3 DPI and 4 DPI, respectively. Remarkably, by 7 DPI, when the positive effects of the other test groups and even the positive control (G4) began to diminish—likely due to chronic effects—G5 continued to show a significantly increased body weight, indicating that the low dose of XG had the most sustained impact. This pattern was further supported by histopathological analysis: at 7 DPI, G5, but not G6 or G7, showed a significantly lower H&E staining score compared to G3 (Figure 3B,C), suggesting a more pronounced reduction in lung inflammation.

**Figure 4:**
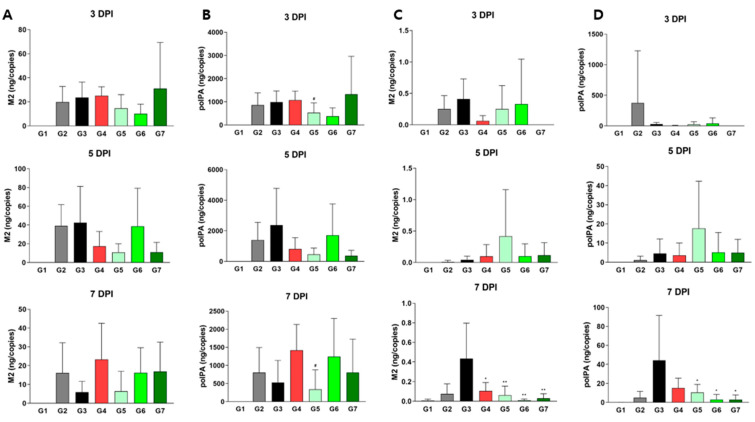
Virus titer quantification in XG-treated mice. Levels of M2 (**A**,**C**) and polPA (**B**,**D**) were measured in lung tissues (**A**,**B**) and nasal cavities (**C**,**D**). Data are presented as means ± s.d.; n = 18 biological replicates. Significance levels are indicated as follows: ^#^ *p* < 0.05 compared to G4 (positive control); ** *p* < 0.01 and * *p* < 0.05 compared to G3 (vehicle control).

To determine whether XG’s physiological and pathological effects were linked directly to viral replication, we measured viral titers in both the lungs and nasal cavity at each stage (Figure 4). In lung tissue, all test groups (G4, G5, and G6) showed minimal differences in the relative levels of M2 and polPA proteins compared to G3; however, G5 had significantly reduced viral titers compared to G4 (Figure 4A,B), reinforcing XG’s superior efficacy over λ-carrageenan in body weight and H&E scores (Figure 3). In the nasal cavity, all test groups and the positive control (G4) exhibited significantly reduced levels of M2 and polPA proteins compared to G3 of DDE at 7 DPI, with particularly strong effects observed in G6 and G7 of DDE (Figure 4C,D). In summary, our findings demonstrate that XG exhibits bona fide antiviral effects at various dosages in vivo, with the low dose showing the most pronounced and sustained benefits.

### 3.3. Synergistic Antiviral Effects of XG and Camostat Against Influenza Viruses In Vitro

**Figure 5:**
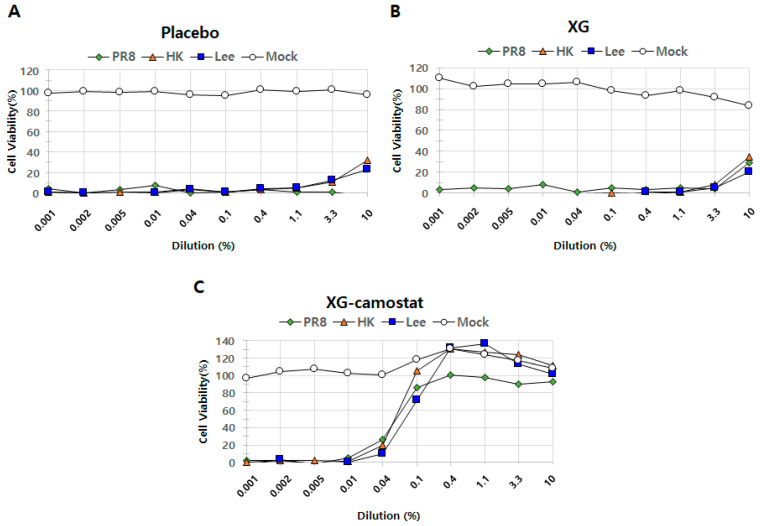
In vitro cell suppression assays demonstrate synergistic antiviral effects of XG and camostat. The relative levels of cell proliferation are shown for the placebo group (**A**), XG treatment alone (**B**), and the combination of XG–camostat (**C**). Quantified data are presented in line graphs for each group, highlighting the impact of the treatments.

Our results so far have demonstrated that XG exhibits antiviral effects at various doses (low, medium, and high) by influencing body weight, lung tissue health, and viral replication. Next, we aimed to determine not only whether XG can suppress the cell amplification of the individual viruses in vitro but also whether XG’s efficacy could be enhanced when combined with an existing antiviral drug, a strategy that could potentially improve drug efficacy against influenza viruses. For this study, we selected camostat, a well-known serine protease inhibitor that blocks TMPRSS2 and has been explored as a potential antiviral drug against COVID-19 [17,18]. Camostat was also chosen due to its oral availability and its potential to act synergistically with XG [18]. We designed three experimental groups to evaluate the antiviral effects of placebo, XG alone, and XG combined with camostat. These groups were tested against three influenza viruses—PR8 (Influenza A H1N1), HK (Influenza A H3N2), and Lee (Influenza B)—with a mock control (vehicle control) included for comparison (Figure 5).

To assess the dynamics in efficacy, we applied a range of dilutions of each testing substance(s) (i.e., of either placebo, XG alone, or XG combined with camostat) from 0.001% to 10.0% across all experimental sets. When converted to mass concentrations, the prepared solutions contain the following amounts of XG and camostat at each concentration: (10%: XG 100 μg/mL, camostat 140 μg/mL; 3.3%: XG 33.3 μg/mL, camostat 46.7 μg/mL; 1.1%: XG 11.1 μg/mL, camostat 15.6 μg/mL; 0.4%: XG 4.0 μg/mL, camostat 5.6 μg/mL; 0.1%: XG 1.0 μg/mL, camostat 1.4 μg/mL; 0.04%: XG 0.4 μg/mL, camostat 0.6 μg/mL; 0.01%: XG 0.1 μg/mL, camostat 0.14 μg/mL; 0.005%: XG 0.05 μg/mL, camostat 0.07 μg/mL; 0.002%: XG 0.02 μg/mL, camostat 0.028 μg/mL) The placebo exhibited minimal antiviral activity across all tested viruses (Figure 5A), while XG alone showed increased antiviral effects, particularly at a dilution rate of 10.0%, where it inhibited 29% of infection compared to −3% in the placebo group (Figure 5B). This finding aligns with the in vivo antiviral effects of XG observed earlier (Figure 1 and Figure 2). Remarkably, the combination of XG with camostat significantly enhanced antiviral activity against PR8 across a range of dilution rates (0.1, 0.4, 1.1, 3.3, and 10.0) (Figure 5C). Additionally, the XG–camostat combination showed a substantial increase in antiviral efficacy against HK and Lee at similar concentrations. The CC50 value of the XG–camostat combination was 10 μM, while the EC50 values were 0.06 μM for PR8, 0.06 μM for HK, and 0.08 μM for Lee. However, further investigation is needed to determine whether this enhancement against HK and Lee is due to a synergistic effect between XG and camostat or solely attributable to camostat. In conclusion, our in vitro assays demonstrate that the combination of XG and camostat has a synergistic effect, particularly against PR8, providing a promising approach for enhancing antiviral treatments.

## 4. Discussion

Due to their multifunctional therapeutic effects and unique biochemical properties, polymers have long been considered potential antiviral agents [29,30]. They are commonly used as carriers to enhance bioavailability and therapeutic efficacy and reduce the required dose of antiviral agents.

In this study, we conducted animal experiments using various known and novel polymers against the H1N1 influenza virus. Among them, XG showed the most promising results and was selected for further investigation. While the polymers used are widely recognized for their beneficial properties, only λ-carrageenan has confirmed direct antiviral effects [15,16]. Fucoidan has been reported to exhibit antioxidant, antihyperglycemic, anti-inflammatory, and anticoagulant effects [7]. In addition to clinical scoring, we assessed its impact on body weight and viral suppression. Bentonite was hypothesized to adsorb contaminants due to its physical properties [11], but the results did not meet expectations. HPMC, previously reported to have antiviral properties [14], also suppressed influenza in our study, but it was not superior to XG or fucoidan in overall performance. Among fucoidan and XG, both of which showed comparable results to the positive control oseltamivir, XG demonstrated better clinical scoring and was selected for further DDE research.

Next, we conducted dose-dependent experiments with XG. Since its antiviral effect was observed at a low concentration (0.1 mg/mL), we incrementally increased the dose to determine the optimal concentration. For comparison, we adjusted the amount of each substance to match the viscosity of λ-carrageenan, an active ingredient in developed antiviral agents, as both XG and λ-carrageenan are viscous polymers. Their viscosity may contribute to viral inhibition by protecting the nasal mucosa. As shown in Figure 3 and Figure 4, XG at 0.005 mg/head resulted in better weight recovery, lower clinical scoring, and reduced viral titers in the nasal cavity and lungs compared to λ-carrageenan at 0.024 mg/head.

Furthermore, we developed a combination treatment by adding camostat, a TMPRSS2 inhibitor that blocks viral entry [27,28], and conducted in vitro experiments. The combination demonstrated enhanced antiviral efficacy, suggesting a synergistic effect. While XG alone prevents or inhibits viral entry, its effectiveness is significantly increased when combined with camostat. This aligns with previous studies highlighting XG’s role in drug delivery systems [31]. The observed synergy between XG and camostat underscores XG’s potential to prevent pandemics or endemics caused by influenza and other viruses. Future studies should explore combinations of XG with various antiviral drugs against diverse viral strains. Our in vivo and in vitro findings provide a strong foundation for future research aimed at combating seasonal influenza threats to both human and animal health.

## 5. Conclusions

Overall, our data suggest that while all polymer-treated groups generally exhibited improved body weight during infection, XG had the most pronounced antiviral effects, both physiologically (body weight) and pathologically (H&E staining and viral titters), making it the most promising candidate as an antiviral polymer. Additionally, the XG–camostat combination showed a substantial increase in antiviral efficacy against all tested viruses in vitro study. The CC50 value of the XG–camostat combination was 10 μM, while the EC50 values were 0.06 μM for PR8, 0.06 μM for HK, and 0.08 μM for Lee. Based on these findings, we plan to further investigate the effects of XG and camostat co-administration in animals infected with various respiratory viruses.

## Figures and Tables

**Table 1:** Composition of experimental groups analyzed in our in vivo screen for antiviral polymers.

Group	Gender	Mouse Number	Infection	Treatment	Dosage (mg/Head)	Volume (µL/Head)	Group Description
G1	Female	1–4 (4)	−	N/A	N/A	20	Normal control
G2	Female	5–19 (15)	+	N/A	N/A	20	Infection control
G3	Female	20–34 (15)	+	Placebo	N/A	20	Vehicle control
G4	Female	35–49 (15)	+	Oseltamivir	0.1	20	Positive control
G5	Female	50–64 (15)	+	λ-Carrageenan	0.024	20	Positive control
G6	Female	65–79 (15)	+	HPMC	0.092	20	Test group
G7	Female	80–94 (15)	+	XG	0.002	20	Test group
G8	Female	95–109 (15)	+	Bentonite	0.18	20	Test group
G9	Female	110–124 (15)	+	Fucoidan	0.16	20	Test group

**Table 2:** Composition of experimental groups analyzed for XG in a dose-dependent manner.

Group	Gender	Mouse Number	Infection	Treatment	Dosage (mg/Head)	Volume (µL/Head)	Group Description
G1	Female	1–4 (4)	−	N/A	N/A	20	Normal control
G2	Female	5–22 (18)	+	N/A	N/A	20	Infection control
G3	Female	23–40 (18)	+	Placebo	N/A	20	Vehicle control
G4	Female	41–58 (18)	+	λ-Carrageenan	0.024	20	Positive control
G5	Female	59–76 (18)	+	XG (low)	0.005	20	Test group
G6	Female	77–94 (18)	+	XG (medium)	0.01	20	Test group
G7	Female	95–112 (18)	+	XG (high)	0.02	20	Test group

## Data Availability

All data are presented in the manuscript and are available from the authors.
