# Correction: Chun et al. Synergistic Antiviral Activity of Xanthan Gum and Camostat Against Influenza Virus Infection. *Viruses* 2025, *17*, 301

**DOI:** 10.3390/v17050605

**Published:** 2025-04-24

**Authors:** Kyeunghwa Chun, Yujeong Na, Byeongyong Kim, Dongjin Lee, Jongseo Choi, Gwanyoung Kim, Sokho Kim, Min-Soo Kim

**Affiliations:** 1 Daewoong Pharmaceutical Co., Ltd., 72, Dugye-ro, Pogok-eup, Cheoin-gu, Yongin-si 17028, Gyeonggi-do, Republic of Korea; khcheon086@daewoong.co.kr (K.C.); 2210494@daewoong.co.kr (Y.N.); 2210565@daewoong.co.kr (B.K.); leedj@daewoong.co.kr (D.L.); 2210302@daewoong.co.kr (J.C.); pharmrich@daewoong.co.kr (G.K.); 2 College of Pharmacy, Pusan National University, 2, Busandaehak-ro 63beon-gil, Geumjeong-gu, Busan 46241, Republic of Korea; 3 Major of Biohealth Regulatory Science, School of Liberal Studies, Kunsan National University, 558 Daehak-ro, Gunsan 54150, Jeollabuk-do, Republic of Korea

## Error in Figure

In the original publication [1], there were mistakes in Figure 3 (Different dosages of XG exhibit suppressive effects on influenza virus infection in vivo) and Figure 5 (In vitro cell suppression assays demonstrate synergistic antiviral effects of XG and camostat) as published.

Upon review, we identified the incorrect placement of the label in Figure 3 and the use of an incorrect graph in Figure 5 of the article. In Figure 3, the label for G4 in Figure 3C was mistakenly written instead of G5. Images in Figure 3C are representative histopathological images from G2, G3, and G5. This error has also been corrected in the Figure legend and caption accordingly. In Figure 5, the graph labeled as “Placebo” in Figure 5A is identical to the “XG” graph in Figure 5B. The graph of the placebo was incorrectly inserted. This error has been corrected by inserting the correct graph of the placebo. We kindly request that the incorrect images be replaced with the corrected versions. The correction only replaced Figures 3 and 5, which does not affect the publication’s conclusion; thus, there is no need to modify the original text.

The corrected Figure 3 and Figure 5 are seen below. The authors state that the scientific conclusions are unaffected. This correction was approved by the Academic Editor. The original publication has also been updated.

## Figures and Tables

**Figure 3:**
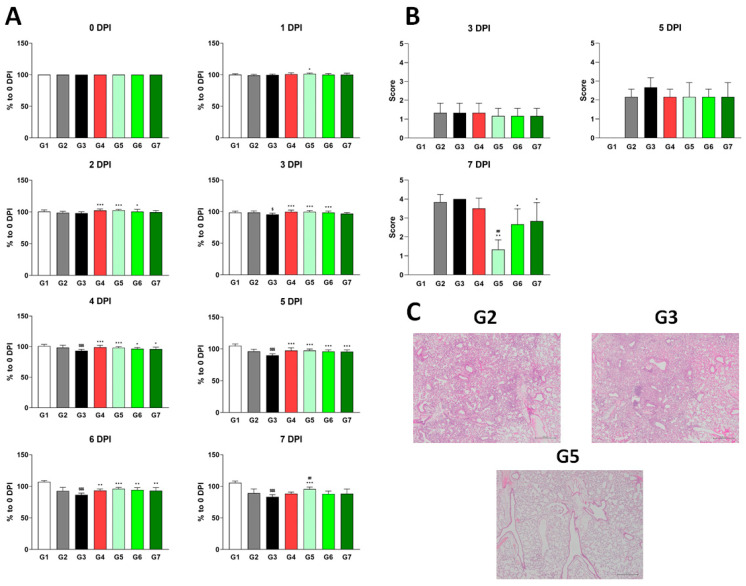
Different dosages of XG exhibit suppressive effects on influenza virus infection in vivo. (**A**) Body weight increases following XG treatment at various dosages. Significance levels are denoted as follows: $$$ *p* < 0.001 and $ *p* < 0.05 compared to G1 (normal control); ## *p* < 0.01 compared to G4 (positive control); ***, **, and * indicate *p* < 0.001, *p* < 0.01, and *p* < 0.05, respectively, compared to G3 (vehicle control). (**B**) Bar graphs represent the relative histopathological scores for each group. (**C**) Representative histopathological images from G2, G3, and G5. Data are presented as means ± s.d.; n = 18 biological replicates per group, except for G1, where four mice were analyzed. Detailed information on the experimental groups is provided in Table 2.

**Figure 5:**
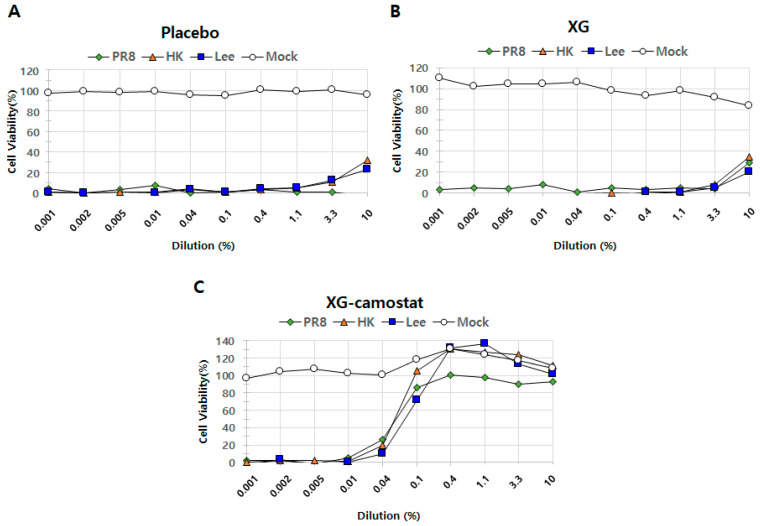
In vitro cell suppression assays demonstrate synergistic antiviral effects of XG and camostat. The relative levels of cell proliferation are shown for the placebo group (**A**), XG treatment alone (**B**), and the combination of XG–camostat (**C**). Quantified data are presented in line graphs for each group, highlighting the impact of the treatments.
